# Supplementary material for: COZOID: contact zone identifier for visual analysis of protein-protein interactions
Source: BMC Bioinformatics. 2018 Apr 6;19:125. doi: 10.1186/s12859-018-2113-6 (PMC5889581; doi:10.1186/s12859-018-2113-6)
Supplement: Supplementary file 2 — Software build. Executable binary file of the software tool. (ZIP 118,784 kb) [file 12859_2018_2113_MOESM2_ESM.zip › cozoid/platform/docs/testng-6.5.1-javadoc.zip!/org/testng/xml/XmlTest.html]

XmlTest (TestNG)


---


|  |  |  |
| --- | --- | --- |
| **Overview** \| **Package** \| **Class** \| **Tree** \| **Deprecated** \| **Index** \| **Help** | |  |
| **PREV CLASS**   **NEXT CLASS** | **FRAMES**    **NO FRAMES**     **All Classes** |  |
| SUMMARY: NESTED \| FIELD \| CONSTR \| METHOD | DETAIL: FIELD \| CONSTR \| METHOD |  |


---


## org.testng.xml Class XmlTest

```
java.lang.Object
  org.testng.xml.XmlTest
```

**All Implemented Interfaces:**: java.io.Serializable, java.lang.Cloneable

---

``` public class XmlTest extends java.lang.Object implements java.io.Serializable, java.lang.Cloneable ```

This class describes the tag <test> in testng.xml.

**Author:**
:   Cedric Beust, Alexandru Popescu

**See Also:**: Serialized Form

---

| **Field Summary** | |
| --- | --- |
| `static int` | `DEFAULT_TIMEOUT_MS` |


**Constructor Summary**

`XmlTest()`

`XmlTest(XmlSuite suite)`

`XmlTest(XmlSuite suite,
int index)`
  
          Constructs a `XmlTest` and adds it to suite's list of tests.


| **Method Summary** | |
| --- | --- |
| `void` | `addExcludedGroup(java.lang.String g)` |
| `void` | `addIncludedGroup(java.lang.String g)` |
| `void` | `addMetaGroup(java.lang.String name, java.util.List<java.lang.String> metaGroup)` |
| `void` | `addParameter(java.lang.String key, java.lang.String value)` |
| `java.lang.Object` | `clone()`             Clone the source `XmlTest` by including: - test attributes - groups definitions - parameters The <classes> sub element is ignored for the moment. |
| `boolean` | `equals(java.lang.Object obj)` |
| `java.lang.Boolean` | `getAllowReturnValues()` |
| `java.util.List<XmlClass>` | `getClasses()` |
| `java.util.List<java.lang.String>` | `getExcludedGroups()` |
| `java.lang.String` | `getExpression()` |
| `boolean` | `getGroupByInstances()` |
| `java.util.List<java.lang.String>` | `getIncludedGroups()` |
| `int` | `getIndex()`             Note that this attribute does not come from the XML file, it's calculated internally and represents the order in which this test tag was found in its <suite> tag. |
| `java.util.List<java.lang.Integer>` | `getInvocationNumbers(java.lang.String method)`             Convenience method to cache the ordering numbers for methods. |
| `java.util.Map<java.lang.String,java.util.List<java.lang.String>>` | `getMetaGroups()` |
| `java.util.List<XmlMethodSelector>` | `getMethodSelectors()` |
| `java.lang.String` | `getName()` |
| `java.util.List<XmlPackage>` | `getPackages()` |
| `java.lang.String` | `getParallel()` |
| `java.lang.String` | `getParameter(java.lang.String name)` |
| `java.util.Map<java.lang.String,java.lang.String>` | `getParameters()`             Returns a merge of the the test parameters and its parent suite parameters. |
| `java.lang.String` | `getPreserveOrder()` |
| `XmlSuite` | `getSuite()`             Returns the suite this test is part of. |
| `java.util.Map<java.lang.String,java.lang.String>` | `getTestParameters()` |
| `int` | `getThreadCount()` |
| `java.lang.String` | `getTimeOut()` |
| `long` | `getTimeOut(long def)` |
| `int` | `getVerbose()` |
| `java.util.List<XmlClass>` | `getXmlClasses()` |
| `java.util.List<XmlPackage>` | `getXmlPackages()` |
| `int` | `hashCode()` |
| `boolean` | `isJUnit()` |
| `void` | `setAllowReturnValues(java.lang.Boolean allowReturnValues)` |
| `void` | `setBeanShellExpression(java.lang.String expression)` |
| `void` | `setClasses(java.util.List<XmlClass> c)` |
| `void` | `setClassNames(java.util.List<XmlClass> classes)`             **Deprecated.** *use setXmlClasses* |
| `void` | `setExcludedGroups(java.util.List<java.lang.String> g)` |
| `void` | `setExpression(java.lang.String expression)` |
| `void` | `setGroupByInstances(boolean f)` |
| `void` | `setIncludedGroups(java.util.List<java.lang.String> g)` |
| `void` | `setJunit(boolean isJUnit)` |
| `void` | `setJUnit(boolean isJUnit)` |
| `void` | `setMetaGroups(java.util.Map<java.lang.String,java.util.List<java.lang.String>> metaGroups)` |
| `void` | `setMethodSelectors(java.util.List<XmlMethodSelector> methodSelectors)` |
| `void` | `setName(java.lang.String name)` |
| `void` | `setPackages(java.util.List<XmlPackage> p)` |
| `void` | `setParallel(java.lang.String parallel)` |
| `void` | `setParameters(java.util.Map<java.lang.String,java.lang.String> parameters)` |
| `void` | `setPreserveOrder(java.lang.String preserveOrder)` |
| `void` | `setSkipFailedInvocationCounts(boolean skip)` |
| `void` | `setSuite(XmlSuite result)` |
| `void` | `setThreadCount(int threadCount)` |
| `void` | `setTimeOut(long timeOut)` |
| `void` | `setVerbose(int v)` |
| `void` | `setXmlClasses(java.util.List<XmlClass> classes)`             Sets the XML Classes. |
| `void` | `setXmlPackages(java.util.List<XmlPackage> packages)` |
| `boolean` | `skipFailedInvocationCounts()` |
| `java.lang.String` | `toString()` |
| `java.lang.String` | `toXml(java.lang.String indent)` |

**Methods inherited from class java.lang.Object**

`finalize, getClass, notify, notifyAll, wait, wait, wait`

**Field Detail**

### DEFAULT\_TIMEOUT\_MS

```
public static int DEFAULT_TIMEOUT_MS
```


**Constructor Detail**

### XmlTest

```
public XmlTest(XmlSuite suite,
               int index)
```

:   Constructs a `XmlTest` and adds it to suite's list of tests.

    **Parameters:**: `suite` - the parent suite.: `index` - the index of this test tag in testng.xml

---


### XmlTest

```
public XmlTest(XmlSuite suite)
```

---


### XmlTest

```
public XmlTest()
```


**Method Detail**

### setXmlPackages

```
public void setXmlPackages(java.util.List<XmlPackage> packages)
```

---


### getXmlPackages

```
public java.util.List<XmlPackage> getXmlPackages()
```

---


### getPackages

```
public java.util.List<XmlPackage> getPackages()
```

---


### setPackages

```
public void setPackages(java.util.List<XmlPackage> p)
```

---


### getMethodSelectors

```
public java.util.List<XmlMethodSelector> getMethodSelectors()
```

---


### setMethodSelectors

```
public void setMethodSelectors(java.util.List<XmlMethodSelector> methodSelectors)
```

---


### getSuite

```
public XmlSuite getSuite()
```

:   Returns the suite this test is part of.

    :   **Returns:**: the suite this test is part of.

---


### getIncludedGroups

```
public java.util.List<java.lang.String> getIncludedGroups()
```

:   **Returns:**: the includedGroups. Note: do not modify the returned value, use `addIncludedGroup(String)`.

---


### setClassNames

```
@Deprecated
public void setClassNames(java.util.List<XmlClass> classes)
```

:   **Deprecated.** *use setXmlClasses*

    :   Sets the XML Classes.

        :   **Parameters:**: `classes` - The classes to set.

---


### getXmlClasses

```
public java.util.List<XmlClass> getXmlClasses()
```

:   **Returns:**: Returns the classes.

---


### getClasses

```
public java.util.List<XmlClass> getClasses()
```

---


### setClasses

```
public void setClasses(java.util.List<XmlClass> c)
```

---


### setXmlClasses

```
public void setXmlClasses(java.util.List<XmlClass> classes)
```

:   Sets the XML Classes.

    :   **Parameters:**: `classes` - The classes to set.

---


### getName

```
public java.lang.String getName()
```

:   **Returns:**: Returns the name.

---


### setName

```
public void setName(java.lang.String name)
```

:   **Parameters:**: `name` - The name to set.

---


### setVerbose

```
public void setVerbose(int v)
```

:   **Parameters:**: `v` -

---


### getThreadCount

```
public int getThreadCount()
```

---


### setThreadCount

```
public void setThreadCount(int threadCount)
```

---


### setIncludedGroups

```
public void setIncludedGroups(java.util.List<java.lang.String> g)
```

:   **Parameters:**: `g` -

---


### setExcludedGroups

```
public void setExcludedGroups(java.util.List<java.lang.String> g)
```

:   **Parameters:**: `g` - The excludedGrousps to set.

---


### getExcludedGroups

```
public java.util.List<java.lang.String> getExcludedGroups()
```

:   **Returns:**: Returns the excludedGroups. Note: do not modify the returned value, use `addExcludedGroup(String)`.

---


### addIncludedGroup

```
public void addIncludedGroup(java.lang.String g)
```

---


### addExcludedGroup

```
public void addExcludedGroup(java.lang.String g)
```

---


### getVerbose

```
public int getVerbose()
```

:   **Returns:**: Returns the verbose.

---


### getGroupByInstances

```
public boolean getGroupByInstances()
```

---


### setGroupByInstances

```
public void setGroupByInstances(boolean f)
```

---


### isJUnit

```
public boolean isJUnit()
```

:   **Returns:**: Returns the isJUnit.

---


### setJUnit

```
public void setJUnit(boolean isJUnit)
```

:   **Parameters:**: `isJUnit` - The isJUnit to set.

---


### setJunit

```
public void setJunit(boolean isJUnit)
```

---


### setSkipFailedInvocationCounts

```
public void setSkipFailedInvocationCounts(boolean skip)
```

---


### skipFailedInvocationCounts

```
public boolean skipFailedInvocationCounts()
```

:   **Returns:**: Returns the isJUnit.

---


### addMetaGroup

```
public void addMetaGroup(java.lang.String name,
                         java.util.List<java.lang.String> metaGroup)
```

---


### setMetaGroups

```
public void setMetaGroups(java.util.Map<java.lang.String,java.util.List<java.lang.String>> metaGroups)
```

---


### getMetaGroups

```
public java.util.Map<java.lang.String,java.util.List<java.lang.String>> getMetaGroups()
```

:   **Returns:**: Returns the metaGroups.

---


### setParameters

```
public void setParameters(java.util.Map<java.lang.String,java.lang.String> parameters)
```

:   **Parameters:**: `parameters` -

---


### addParameter

```
public void addParameter(java.lang.String key,
                         java.lang.String value)
```

---


### getParameter

```
public java.lang.String getParameter(java.lang.String name)
```

---


### getParameters

```
public java.util.Map<java.lang.String,java.lang.String> getParameters()
```

:   Returns a merge of the the test parameters and its parent suite parameters. Test parameters
    have precedence over suite parameters.

    :   **Returns:**: a merge of the the test parameters and its parent suite parameters.

---


### getTestParameters

```
public java.util.Map<java.lang.String,java.lang.String> getTestParameters()
```

:   **Returns:**: the parameters defined on this tag only

---


### setParallel

```
public void setParallel(java.lang.String parallel)
```

---


### getParallel

```
public java.lang.String getParallel()
```

---


### getTimeOut

```
public java.lang.String getTimeOut()
```

---


### getTimeOut

```
public long getTimeOut(long def)
```

---


### setTimeOut

```
public void setTimeOut(long timeOut)
```

---


### setExpression

```
public void setExpression(java.lang.String expression)
```

---


### setBeanShellExpression

```
public void setBeanShellExpression(java.lang.String expression)
```

---


### getExpression

```
public java.lang.String getExpression()
```

---


### toXml

```
public java.lang.String toXml(java.lang.String indent)
```

---


### toString

```
public java.lang.String toString()
```

:   **Overrides:**: `toString` in class `java.lang.Object`

---


### clone

```
public java.lang.Object clone()
```

:   Clone the source `XmlTest` by including:
    - test attributes
    - groups definitions
    - parameters
    The <classes> sub element is ignored for the moment.

    :   **Overrides:**: `clone` in class `java.lang.Object`
    :   **Returns:**: a clone of the current XmlTest

---


### getInvocationNumbers

```
public java.util.List<java.lang.Integer> getInvocationNumbers(java.lang.String method)
```

:   Convenience method to cache the ordering numbers for methods.

---


### setPreserveOrder

```
public void setPreserveOrder(java.lang.String preserveOrder)
```

---


### getPreserveOrder

```
public java.lang.String getPreserveOrder()
```

---


### setSuite

```
public void setSuite(XmlSuite result)
```

---


### getAllowReturnValues

```
public java.lang.Boolean getAllowReturnValues()
```

---


### setAllowReturnValues

```
public void setAllowReturnValues(java.lang.Boolean allowReturnValues)
```

---


### getIndex

```
public int getIndex()
```

:   Note that this attribute does not come from the XML file, it's calculated
    internally and represents the order in which this test tag was found in its
    <suite> tag. It's used to calculate the ordering of the tests
    when preserve-test-order is true.

---


### hashCode

```
public int hashCode()
```

:   **Overrides:**: `hashCode` in class `java.lang.Object`

---


### equals

```
public boolean equals(java.lang.Object obj)
```

:   **Overrides:**: `equals` in class `java.lang.Object`


---


|  |  |  |
| --- | --- | --- |
| **Overview** \| **Package** \| **Class** \| **Tree** \| **Deprecated** \| **Index** \| **Help** | |  |
| **PREV CLASS**   **NEXT CLASS** | **FRAMES**    **NO FRAMES**     **All Classes** |  |
| SUMMARY: NESTED \| FIELD \| CONSTR \| METHOD | DETAIL: FIELD \| CONSTR \| METHOD |  |


---
